# A Dietary Intervention to Lower Serum Levels of IGF-I in *BRCA* Mutation Carriers

**DOI:** 10.3390/cancers10090309

**Published:** 2018-09-04

**Authors:** Patrizia Pasanisi, Eleonora Bruno, Elisabetta Venturelli, Daniele Morelli, Andreina Oliverio, Ivan Baldassari, Francesca Rovera, Giovanna Iula, Monica Taborelli, Bernard Peissel, Jacopo Azzollini, Siranoush Manoukian

**Affiliations:** 1 Unit of Epidemiology and Prevention, Fondazione IRCCS Istituto Nazionale dei Tumori di Milano, 20133 Milano, Italy; patrizia.pasanisi@istitutotumori.mi.it (P.P.); elisabetta.venturelli@istitutotumori.mi.it (E.V.); andreina.oliverio@istitutotumori.mi.it (A.O.); ivan.baldassari@istitutotumori.mi.it (I.B.); 2 Department of Biomedical Sciences for Health, University of Milan, 20133 Milano, Italy; 3 Unit of Laboratory Medicine, Fondazione IRCCS Istituto Nazionale dei Tumori di Milano, 20133 Milano, Italy; daniele.morelli@istitutotumori.mi.it; 4 Senology Research Center—1st Division of Surgery, Department of Surgical and Morphological Sciences, University of Insubria, 21100 Varese, Italy; francesca.rovera@uninsubria.it; 5 Senology Research Center—ASST Spedali Civili di Brescia, 25123 Brescia, Italy; iula.giovanna@gmail.com; 6 Genetic Counselling Service, Institute of Oncology of Southern Switzerland (IOSI), 6500 Bellinzona, Switzerland; Monica.Taborelli@eoc.ch; 7 Unit of Medical Genetics, Fondazione IRCCS Istituto Nazionale dei Tumori di Milano, 20133 Milano, Italy; bernard.peissel@istitutotumori.mi.it (B.P.); jacopo.azzolini@istitutotumori.mi.it (J.A.); siranoush.manoukian@istitutotumori.mi.it (S.M.)

**Keywords:** *BRCA* genes, mutation carriers, IGF-I, diet, penetrance

## Abstract

Background: Insulin-like growth factor I (IGF-I) and other markers of insulin resistance (IRm) might influence the penetrance of *BRCA* gene mutation. In a demonstration project on *BRCA* mutation carriers we tested the effect of the ‘Mediterranean diet’, with moderate protein restriction, on serum levels of IGF-I and IRm. Methods: *BRCA* mutation carriers, with or without breast cancer, aged 18–70 years and without metastases were eligible. After the baseline examinations, women were randomized to an active dietary intervention or to a control group. The intervention group attended six full days of life-style intervention activities (cookery classes followed by lunch, sessions of walking for 45 min and nutritional conferences) over the next six months. Results: 213 *BRCA* mutation carriers completed the six-month study. Women in the intervention group (110) showed major changes in all the parameters under study. They significantly lost weight (*p* < 0.001), fat mass (*p* = 0.002), with reduced hip circumference (*p* = 0.01), triglycerides (*p* = 0.02) and IGF-I (*p* = 0.02) compared with controls. They also had a significantly higher levels of insulin-like growth factor-binding protein 3 (IGFI-BP3) (*p* = 0.03) and a lower IGF-I/IGFI-BP3 ratio (*p* = 0.04). The reduction of serum levels of IGF-I was significantly associated with the reduction in the consumption of animal products (*p* = 0.04). Conclusions: Women in the intervention group showed significant improvements in IGF-I and in other IRm that might influence the penetrance of BRCA mutations.

## 1. Introduction

Up to 5% of breast cancers (BC) and 10% of ovarian cancers may be associated with autosomal dominant *BRCA* gene pathogenic variants (mutations) [1,2]. The lifetime cumulative risk (penetrance) of BC associated with *BRCA* mutations is probably of the order of 50%, ranging from about 80% in high-risk families to around 40% in population-based studies [3,4,5,6,7,8]. However, since not all *BRCA* mutation carriers develop cancer, the penetrance of this genetic defect may be regulated by other genetic or environmental factors such as diet, metabolic, and growth factors. Markers of insulin resistance (IRm), such as high serum levels of insulin, insulin-like growth factor I (IGF-I), glucose, and the metabolic syndrome are associated with an increased risk of sporadic BC and may also affect BC prognosis [9,10,11,12,13]. We hypothesized that IRm might also be important for hereditary BC.

Women at high genetic risk of BC have an elevated prevalence of IRm and metabolic syndrome [14]. Observational data indicated that central obesity and high body weight are associated with a greater risk of BC [15] in women from high-risk families than in women with no family history of BC. High energy intake, which is usually associated with higher levels of IGF-I, was significantly associated with BC penetrance in *BRCA* mutation carriers, while adulthood reduction in body weight appeared as an important protective factor [16]. Obesity and diabetes are associated with BC risk in *BRCA* women [17]. A recent study on 6052 *BRCA* mutation carriers showed that *BRCA* women with BC more frequently developed a type-2 diabetes. The authors suggested that the pre-diabetic condition, when the levels of insulin and growth factors are typically very high, increases the risk of breast tumours in carriers of the *BRCA* mutation [17].

Our multinational case-only study (C.O.S.) [18,19,20] on 3000 young women (<40 years of age) showed a significant positive association with high consumption of milk among women with a substantial probability of *BRCA* mutation [19,21]. Milk directly stimulates insulin production or release [22], and it is associated with higher plasma levels of IGF-I [23]. A functional interaction between the *BRCA* genes and IGF-I system was confirmed by mechanistic studies. The IGF-I signalling pathway regulates *BRCA1* gene expression and wild-type *BRCA1* strongly reduces the synthesis of IGF-I receptors [24,25,26]. A recent review emphasized the convergence of IGF-I-mediated cell survival, proliferative pathways, and *BRCA*-mediated tumor protective pathway. The mutation in the *BRCA1* gene causes the loss of its tumor protective action, thus leading to constitutive activation of the IGF-IR signalling pathway relevant for breast, ovarian, and other types of cancer [27]. In line with this, our case-control analysis of 308 high genetic risk women found that high serum levels of IGF-I were associated with significantly increased penetrance [28].

The regulation of IGF-I by diet is complex. More than 20 years ago Thissen et al. showed that the plasma concentration of IGF-I drops steeply after a few days of fasting, due to the development of growth hormone (GH) resistance [29]. In the same years, studies on rodents showed that long-term moderate calorie restriction also lowered IGF-I concentrations by 20–40% [30,31]. Several subsequent studies, however, including one recent randomized trial [32] have failed to show any reduction of IGF-I in humans after long-term calorie restriction. GH sensitivity is also regulated by insulin. However, our previous randomized controlled trials on post-menopausal women, the DIANA (Diet and Androgens) trials, found that a Mediterranean/macrobiotic diet, without protein restriction, lowered insulin and serum levels of sex hormones, raised sex hormone-binding globulin (SHBG) and the insulin-like growth factor-binding proteins (IGFBP)-1 and -2, but did not reduce IGF-I levels [33,34,35].

In humans, calorie restriction alone does not seem to significantly lower IGF-I; protein restriction is also required [36]. Men and women belonging to the Calorie Restriction Society, in fact, who consume a fairly large amount of protein, had lower levels of several biomarkers of cardiovascular risk, but not of IGF-I [36]. IGF-I, however, was markedly reduced in raw food vegetarians, who have very low protein consumption (0.9 g/kg body weight) [37]. Fontana et al. [38] persuaded six members of the Calorie Restriction Society to reduce their protein intake from 24% to about 10% for three weeks. This resulted in plasma IGF-I falling from 194 ng/mL to 152 ng/mL. Therefore, protein restriction might be effective in reducing IGF-I.

As part of a demonstration project on *BRCA* mutation carriers aimed at clarifying the role of lifestyle and metabolic factors in *BRCA* mutations penetrance, we are conducting a randomized controlled trial to test whether a dietary intervention based on the ‘Mediterranean diet’ (MedDiet) with moderate protein restriction significantly reduces IGF-I and IRm [21]. Preliminary findings from this trial indicated significantly higher adherence to the MedDiet among the women in the intervention group and suggested an inverse relation between the parameters of metabolic syndrome and adherence to the MedDiet [39].

This paper reports the findings of the “intention-to-treat analysis” for the primary endpoints of the trial, i.e., the effect of the dietary intervention on serum levels of IGF-I and other IRm, and of the effect of specific dietary components on the markers of interest.

## 2. Results

**Figure 1:**
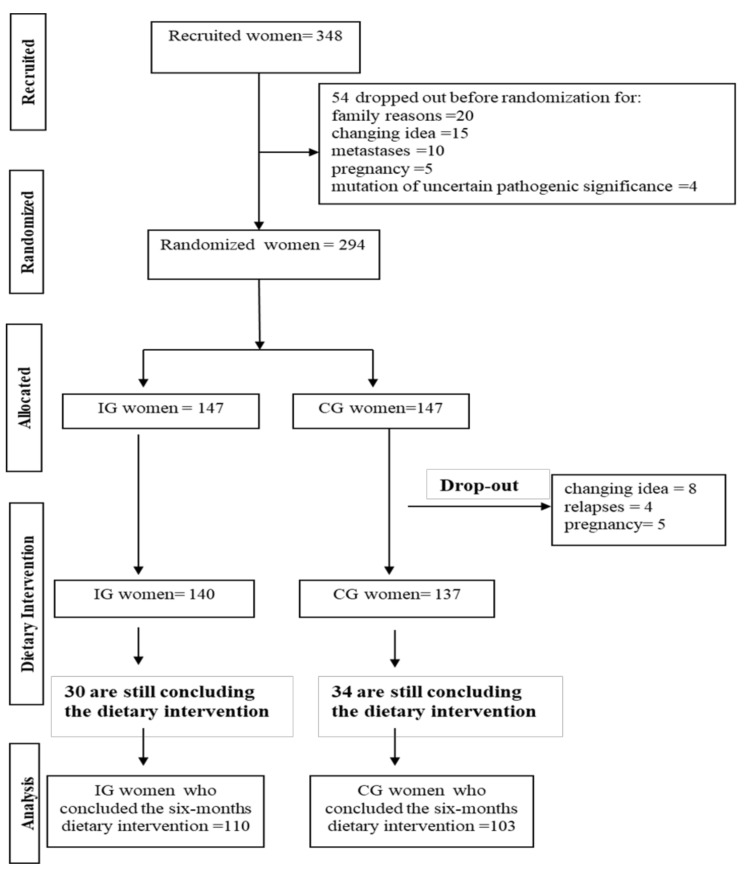
Flowchart of the trial. IG: Intervention Group; CG: Control Group.

Figure 1 represents the flowchart of the trial. At the time of writing, 348 *BRCA* mutated women have signed informed consent. Fifty-four women dropped out the study before the baseline examinations and randomization. Among these, twenty dropped out for family reasons, fifteen changed their mind, ten presented metastases at the baseline clinical visit and five were pregnant. Four others reported a *BRCA* mutation of uncertain pathogenic significance, and we decided not to include them. Therefore, 294 women were properly randomized in an intervention group (IG = 147) or in a control group (CG = 147). After randomization, four women became pregnant, five relapsed and eight decided to drop out at the beginning of the dietary intervention. Another 64 women are still concluding the six-month dietary intervention.

The present results refer to the 213 women (110 in the IG and 103 in the CG) who have already concluded the dietary intervention trial. Complete baseline and six-month dietary, anthropometric, metabolic and hormonal data are available for all of them. Among these women, 123 women presented a *BRCA1* mutation, 88 a *BRCA2* mutation and two both mutations. The 68% of the women in IG and 65% of controls had a previous diagnosis of BC (*p* = 0.63).

The general characteristics of the trial population by randomization group are reported in Table 1. The infiltrating duct BC was the most frequent histological type (80% on average), and Estrogen Receceptor (ER) negative tumors were slightly higher represented. Control women showed a not significant higher frequency of axillary node metastasis (*p* = 0.38) and G3 tumours (*p* = 0.56) (Table 1).

Regarding to anamnestic data, both randomized groups showed similar age at menarche, age at first live birth and levels of education. 73.6% of women of the intervention and 65.5% of the control group were in menopause at the time of recruitment (*p* = 0.19), but only 11% (in the IG) and 7% (in the CG) were in natural menopause. Intervention women were lesser oral contraceptive users (*p* = 0.33) and smokers in the past (*p* = 0.66), and presented a slightly higher frequency of number of children (*p* = 0.49) (Table 1).

As regards the metabolic and anthropometric parameters, at baseline the two groups were fairly homogeneous. Intervention women were slightly higher (*p* = 0.09) and thinner (*p* = 0.20), but showed higher levels of triglycerides (*p* = 0.09) and insulin (*p* = 0.11), and lower levels of High Density Lipoprotein (HDL) cholesterol (*p* = 0.19) [39].

Table 2 reports the baseline frequency of consumption of selected food or food groups (by randomization group) according to the 24-h food frequency diaries. Food and food groups consumption is described according to three categories (0 = not consumed; 1 = 1 time/day; >1 = more than 1 time/day). The two groups were substantially homogeneous as regards their food consumptions. At baseline, IG showed a slightly higher consumption of legumes, added sugars and alcoholic drinks compared to CG (Table 2).

The before/after analysis indicated that both groups significantly improved most of the metabolic parameters, but intervention women showed changes with major impact for health in all the parameters under study (Table 3). Furthermore, control women did not significantly reduce total cholesterol (*p* = 0.14) and their triglycerides - a factor of the metabolic syndrome—rose significantly (*p* = 0.004). Only intervention women had significant reductions of body weight, Body Mass Index (BMI), and fat mass, while lean mass and muscle mass did not substantially change.

At the end of the six-month dietary intervention the CG had no substantial changes in IGF-I (−2.5 ng/mL, *p* = 0.57) while the IG had a significant reduction (−15.0 ng/mL, *p* < 0.001). Both groups had significant reductions in insulin (−6.4 μIU/mL, p = 0.001 in the CG, and −10.5 μIU/mL, *p* < 0.001 in the IG). The CG had a significant reduction in Insulin-like Growth Factor-Binding Protein 3 (IGFI-BP3) (−0.24 μg/mL, *p* = 0.01) while the IG showed a non-significant increase (+0.06 μg/mL, *p* = 0.51).

RM-ANOVA suggested significant interactions for IGF-I, IGFI-BP3 and some body composition parameters. This means that the two groups present different patterns of change in IGF-I and other IRm over time. The “delta” analysis of differences between the two groups (intention-to-treat analysis), controlling for age and BMI at baseline, showed that intervention women lost weight (*p* < 0.001) and fat mass (*p* = 0.002), and lowered hip circumference (*p* = 0.01) and triglycerides (*p* = 0.02) significantly more than controls. For IGF-I and the other hormonal parameters, the “delta” analysis of differences between the groups showed that intervention women significantly lowered IGF-I (*p* = 0.02) and significantly increased IGFI-BP3 (*p* = 0.03) with respect to controls. Intervention women also showed significant improvement in the IGF-I/IGFI-BP3 ratio (0.04) (Table 4). On repeating the analysis, adjusting for weight change too, the results did not differ.

Table 5 shows the proportion of women who worsened, did not change, or improved their food consumptions (data from 24-h food frequency diaries) between baseline and the end of the six-month dietary intervention by randomization group. Both groups showed some improvements in food consumptions but women in the IG showed changes of major impact. Compared to CG, IG significantly improved the consumption of the recommended food, i.e., whole cereals (*p* = 0.02) and whole grain products (*p* = 0.01), and significantly reduced the consumption of refined food (*p* = 0.01), dairy products (*p* = 0.01), and animal products (*p* = 0.04).

The reduction in animal products consumption (especially in milk and dairy products) was directly correlated to the reduction of IGF-I levels. The multiple regression model reported a significant association between the reduction of animal products consumption and the reduction in serum levels of IGF-I (*p* = 0.04), controlling for age, BMI, and weight change. A borderline significant association was also observed with the reduction of refined products and added sugars (*p* = 0.06).

No other significant association appeared between IGF-I or other IRm and change in consumptions of other food groups.

## 3. Discussion

The primary endpoints of our dietary randomized controlled trial indicate that *BRCA* mutation carriers in the intervention group enjoyed significant improvements in serum levels of IGF-I, IGFI-BP3 and IGF-I/IGFI-BP3 ratio compared to the control women. Previous trials have showed that both calorie restriction and an insulin-lowering diet reduce the bioavailability of IGF-I by increasing the liver synthesis of IGFBP 1 and 2, but do not reduce serum levels of IGF-I [32,35]. In a small physiologic study on six subjects practicing severe calorie restriction, Fontana el al. [38] showed that reducing protein intake from 1.6 to 0.9 g per kg per day markedly lowered the IGF-I concentration, suggesting that calorie restriction does not reduce IGF-I unless protein intake is reduced as well. The present larger trial on *BRCA* mutation carriers now corroborates this. We succeeded in lowering serum levels of IGF-I by a comprehensive change in diet with moderate calorie and protein restriction, based on the traditional Mediterranean diet. Our results from the analysis of 24-h food frequency diaries on food consumptions of the previous day support that IG strongly increased satiating food (whole grain products) and reduced high calorie-dense food (sugars and refined products), dairy and total animal products thus experiencing an higher reduction in body weight, fat mass and IGF-I levels. Our results suggest a direct relationship between the reduction in animal products consumption (especially in milk and dairy products), as requested by our dietary recommendations, and the reduction in serum levels of IGF-I. Compared with women whose consumption of animal products did not change or got worse, women who decreased their consumption achieved the greater reduction of IGF-I. The reduction of IGF-I was significantly related to the randomization group (dietary intervention) and to the reduction of animal products consumption.

Another interesting finding of our trial is that the effects on IGF-I, IGFI-BP3 and the IGF-I /IGFI-BP3 ratio persisted significantly after adjustment for weight change, suggesting they were due more to the global composition of the diet than to the change of calorie intake.

We believe that the reduction of IGF-I might be particularly important for *BRCA* mutation carriers. Lifestyle factors linked to insulin resistance such as high serum levels of insulin and IGF-I, abdominal adiposity, high energy intake, milk consumption and low levels of physical activity have in fact been associated with higher penetrance of BC in *BRCA* mutation carriers [21,28]. Growing knowledge about *BRCA* genes, alongside the introduction of predictive genetic tests, has made it possible to identify women at high risk of BC and ovarian cancer. Current preventive options for these women include surveillance programs aimed at early diagnosis, risk-reducing surgery (bilateral prophylactic mastectomy and annessiectomy), while non-surgical options for prevention (e.g., chemo-prevention with drugs that interrupt the signals mediated by estrogen) are not yet firmly established. Adjuvant treatment with tamoxifen has been associated with a lower incidence of contra-lateral BC in *BRCA* positive patients [40,41]. However, this drug seems equally effective in *BRCA2* carriers (who mainly have estrogen receptor-positive tumors) and those with *BRCA1* (who mainly have estrogen receptor-negative tumors), suggesting that the mechanism of prevention may be partly mediated by lowering the levels of IGF-I [42].

A limit of our study is that, at this moment, we are not able to quantify how, the reduction in IGF-I levels that we observed in the IG, might be translated in a change of *BRCA* BC risk. Several studies tested the association between IGF-I and the risk of “sporadic” BC [12]. By considering the relative risks reported in the ORDET study from our group [43], a 10% reduction of IGF-I levels (as we obtained in our trial) corresponds to a reduction in the risk of BC in the order of 10–20%. Unfortunately, no studies exist in this context on *BRCA* mutation carriers. We think that our prospective randomized trial would help to understand the impact of lifestyle interventions in prevention of cancer as well as on prognosis also in *BRCA* carriers.

There is growing interest in nutrition, so it is hardly surprising that *BRCA* mutation carriers continue to inform themselves about what they should or should not eat in order to limit their risk of disease [44,45]. Might primary prevention options such as lifestyle changes be useful for *BRCA* mutation carriers? The present results and our previous findings [39] suggest that women with *BRCA* mutations may gain from dietary recommendations, with significant improvements in their IRm and metabolic and anthropometric parameters.

In our trial, the control women also showed some improvements in anthropometric, metabolic and hormonal parameters. They had significant reductions in waist and hip circumferences, LDL cholesterol, plasma glucose and serum insulin. According to our trial design all women, at the recruitment day, attended a conference in which the Word Cancer Research Fund/ American Institute for Cancer Research (WCRF/AICR) recommendations for cancer prevention are explained and received a standard meal that respects WCRF principles [46]. These lifestyle recommendations and the special attention of high genetic risk women to the benefits of a healthy diet led the control group to make some adjustments in their dietary habits. However, the intervention women enjoyed improvements with major impact for health in all the parameters under study, and the controls’ changes did not affect body weight, body composition, triglycerides or IGF-I: calorie and protein restriction is required to reduce IGF-I.

Our findings demonstrate that women carrying *BRCA* mutations, if properly instructed, are capable of substantially improve their dietary style, ameliorate their metabolic parameters, and reduce the serum concentration and the bioavailability of IGF-I, possibly a major determinant of the penetrance of *BRCA* mutations.

Right now another randomized controlled trial in *BRCA* mutation carriers is ongoing in Germany, with the aim of demonstrating improvements in physical fitness, nutritional behaviour and BMI in the intervention arm [47]. Both studies will follow-up the participants to assess whether IGF-I, IRm, and their change over time affect subsequent BC incidence and recurrences. We are confident that larger numbers of participants and close follow-up of the cohorts should substantially boost our understanding of the “environmental” modulators of *BRCA* penetrance.

## 4. Subjects and Methods

### 4.1. Women

The main details of the trial have already been described [39]. Briefly, eligible study subjects were women with proved deleterious *BRCA* mutations, aged 18–70, with or without BC, and without metastases. Unaffected *BRCA* mutation carriers with bilateral prophylactic mastectomy were not included in the cohort.

Recruitment was conducted with the collaboration of general practitioners, geneticists and oncologists, and by advertising the trial in the mass media. The study was approved by the Ethics Committee of the Fondazione IRCCS Istituto Nazionale dei Tumori, Milan (ethical code: INT106/13, date of approval 22 July 2013). The trial was retrospectively registered at www.clinicaltrial.gov (reference: NCT03066856) on 28 February 2017.

Participants in the trial were fully informed about the aim and design of the study, signed an informed consent and were asked:

- to complete the EPIC-Italy questionnaire on their medical history and major cancer risk factors;
- to attend an anthropometric visit at baseline and at the end of the trial;
- to give a 20-mL blood sample to measure IGF-I, insulin and other IRm at baseline and at the end of the trial;
- to provide information on their health and any intervening diseases
- to complete at baseline and at the end of the trial questionnaires on adherence to the MedDiet (MEDAS) [48] and 24-h recalls of the previous day’s food intake.
- to agree to participate in the life-style intervention in case of randomization to the intervention group.

All participants received at baseline the World Cancer Research Fund recommendations for the dietary prevention of cancer [46]. After baseline examinations, women were randomized to an active dietary IG or to a CG that carried on following the baseline recommendations. The IG was invited to attend six full days of life-style intervention activities over the next six months. These activities included six cookery classes followed by lunch, six physical activity sessions (walking for 45 min) and six conferences.

### 4.2. Anthropometric Visit

Women were requested to attend a clinical visit to obtain anthropometric and body composition measurements (lean, fat, and muscle mass using a body segmental impedance balance, Tanita BC-418, (Tanita Corporation, Tokyo, Japan). Height and body weight were measured without shoes and heavy clothes. Waist circumference was measured with a measuring tape at natural waist or, if not identifiable, at the midpoint between the iliac crest and the lower rib. Hip circumference was measured at the level of the throcanters. Blood pressure was measured using an electronic sphygmomanometer.

### 4.3. Dietary Data Collection

All *BRCA* women completed a 24-h food frequency diary on their consumptions of the previous day at baseline and at the end of the six-month dietary intervention. The diary contained a list of 65 food items organized in the following six food groups:

1) drinks, milk, dairy products
2) sweets and confectionery
3) bread and cereals
4) meat, fish, eggs and meat substitutes
5) legumes, vegetables, fresh and dried fruit
6) sauces, animal and vegetable fats

The diary did not include information on portion size or weight, nor on recipes. Women had to indicate only whether, the previous day, they had or not eaten the specified food at breakfast, lunch, dinner and breaks.

### 4.4. Dietary Intervention

The dietary intervention consisted of six full days of nutritional activities over a six months period. These activities included cookery classes followed by common meals, and nutritional conferences. Women in the IG received handouts with recipes, illustrations of the food portions, particularly of food with high protein content and ad hoc dietary recommendations.

The aim of the trial was to reduce serum levels of IGF-I, insulin and other IRm by using a MedDiet with moderate calorie and protein restriction. In detail, recommendations for women in the IG included:

- reducing protein intake down to 10–12% of total calorie intake, mainly milk and animal protein (except fish). The nutritional content of the menus provided during cookery classes, meals and recipes that participants receive as information material was assessed at the beginning of the study;
- reducing calorie intake, preferring highly satiating food (unrefined cereals, legumes and vegetables);
- reducing high glycemic index food, such as refined flours, potatoes, white rice, corn flakes, and high insulinemic food, such as sugar and milk;
- reducing sources of saturated fat (red and processed meat, milk and dairy products);
- eating mostly food of plant origin, with a wide variety of seasonal products.

Participants were encouraged to increase the consumption of whole-grain rice, barley, millet, oat, spelt, quinoa and buckwheat, legumes (any type including soy products), vegetables (any except potatoes) and to prefer unrefined vegetable fats, such as olive oil, nuts and oleaginous seeds. They were taught how to prepare traditional Mediterranean and macrobiotic dishes based on grain, pasta and legumes, seasoned with vegetable sauces and little fat; cakes and biscuits without sugar, milk, butter, and refined flour, using instead dried fruit, such as raisins and apricots, oleaginous seeds, soy milk, cereal flakes or unrefined flour.

We frequently used dietary sources of anti-inflammatory items such as small oily fish, seaweeds, turmeric, green tea, olive oil, wild berries and other quercetin-rich vegetables, such as capers, apples, onions - especially red onions, broccoli and other leafy green vegetables.

### 4.5. Laboratory Methods

Blood samples, at baseline and at the end of the six-month dietary intervention, were collected for all participants 120 min after a standard meal (miso soup, brown rice seasoned with sesame seeds and salt, vegetables and a small portion of legumes—e.g., 50 g before cooking).

Women were asked to give 20 mL of blood; we prepared eight samples of serum (4), plasma (2), red blood cells (1) and buffy coat (1). The samples were aliquoted and stored at −80 °C.

Plasma glucose, triglycerides, total, LDL and HDL cholesterol, and insulin were measured using routine laboratory techniques. Serum IGF-I was measured using commercial radioimmunoassay kits from Biosource (Nivelles, Belgium). The intra- and inter-assay coefficients of variation were respectively 2.8% and 5.3% for a mean IGF-I level of 304 ng/mL. The technicians analyzing the serum samples were blinded to the case or control status of the patients.

### 4.6. Statistical Analysis

We established the trial sample size assuming that the planned dietary intervention might lower IGF-I by about 15% on average. Enrolling 78 women per group (with the control group receiving only general dietary recommendations) would give about 90% power with a two-sided alpha 0.05 to detect this difference.

The distributions of each parameter, tested for normality by a graphic method, were normally distributed. The parameter results were expressed as mean ± standard deviation (SD).

Body mass index (BMI) was defined as body weight/height squared (BMI = kg/m^2^).

At baseline, the means of continuous variables in the intervention group were compared with the control group using Student’s t test. A χ^2^ test was used to compare frequencies.

We described data from 24-h diaries by single food and food groups. We generated food group variables by summing single food items: added sugars (white sugar + brown sugar + malt); refined food (white bread + white rice + egg noodles + corn flakes + sweetened muesli + biscuits); whole-grain products (whole bread + whole rice + other whole grain cereals + unsweetened muesli + oat flakes); whole cereals (whole rice + other whole grain cereals ); legumes and soy product (legumes + tofu/tempeh); dairy products (milk + dairy); animal products (milk + dairy + meat + eggs). Food and food groups consumption was described according to three categories (0 = not consumed; 1 = 1 time/day; >1 = more than 1 time/day), and χ ^2^ test was used to compare the distribution of women of IG and CG in these categories.

A two-sample t test was used to compare baseline and six-month measurements in the two groups. We used ANOVA for repeated measures (RM-ANOVA) to check for interactions between the two independent variables (group and time) on the dependent variable (factors under study). The model took into account time as the within-subjects factor and group as between-subjects factor. When there was a significant interaction between time and group, we analyzed the magnitude of changes in IGF-I, insulin, IGFI-BP3, anthropometric and metabolic variables using the difference (*delta*, *Δ*) between the end of the study and baseline values for each woman in the two groups, and controlled for quintiles of age, quintiles of BMI at baseline and quintiles of weight change. As regards dietary variables, we analyzed the magnitude of *Δ*food consumptions comparing the proportion of women who changed, after the six-months intervention, their food consumptions according to three different categories (worsening, no change, improving).

The association between changes in IGF-I and other IRm and changes in food consumptions was studied with a multiple regression model, including age (quintiles), BMI at baseline (quintiles), weight change (in quintiles) and randomization group as model covariates.

A *p*-value of <0.05 was taken as significant. All statistical tests were two-sided. Analyses were done using the STATA 12 statistical package (StataCorp LP College Station, TX, USA).

## 5. Conclusions

Human carcinogenesis is a complex non-linear process that depends on a large number of interconnected genetic and metabolic pathways which need to be tackled with a many-faceted preventive strategy. These are the first results about the effect of a dietary intervention on potential modulators of *BRCA* penetrance. The women with *BRCA* mutations randomized in the intervention group had significantly lower serum levels of IGF-I, higher levels of IGFI-BP3 and a better IGF-I/IGFI-BP3 ratio, and enjoyed significant improvements in their dietary, metabolic and anthropometric parameters compared to controls. We feel there is time to offer recommendations to genetic high-risk women regarding their lifestyle choices.

## Figures and Tables

**Table 1:** Baseline characteristics of the study population (213 women).

Mean ± S.D.	Intervention (110)	Control (103)
Age (yrs) BMI Menarche (yrs) Age at first live birth (yrs)	48.0 ± 10.3 24.0 ± 4.8 12.7 ± 1.2 28.3 ± 5.3	45.4 ± 10.3 24.2 ± 4.9 12.3 ± 1.5 29.2 ± 4.7
(%)		
Infiltrating duct BC	78	82
ER-negative	53.6	55.3
Axillary node metastasis	31	38
Grading		
G1 G2 G3	6.1 34.7 59.2	9.1 22.7 68.1
Education		
First level Second level Third level	17.3 45.5 37.3	17.5 42.7 38.8
Pregnancy (yes)	74.3	73.4
Number of children		
≤2 3 >3	35.8 50.6 13.6	35.3 56.6 6.9
Menopause Natural menopause Oral contraceptive in the past Smoke in the past	65.4 11 69.7 26.6	73.6 7 75.7 29.1

**Table 2:** Baseline frequency of consumption of selected food or food groups (213 women).

Food Frequency Consumption (%)	Intervention Group (110)			Control Group (103)			*p* *
	0 (%)	1 (%)	>1 (%)	0 (%)	1 (%)	>1 (%)	
Added sugars	53.7	25	21.3	41.9	36.2	21.9	0.15
Refined food	19.4	35.2	45.4	17.2	29.5	53.3	0.50
Whole-grain products	33.3	38	28.7	37.1	28.6	34.3	0.34
Whole-grain cereals	77.8	19.4	2.8	81.9	13.3	4.8	0.39
Legumes /Soy products	62.3	31.5	6.2	64.8	34.3	0.9	0.09
Dairy products	39.8	39.7	20.5	39.1	43.8	17.1	0.77
Meat	48.3	35.2	16.5	48.1	29.1	22.8	0.15
Animal products	33.3	41.7	25	27.6	40	32.4	0.44
Vegetables	4.6	20.4	75	2.9	21.9	75.2	0.78
Fruits	23.1	26.9	50	20	37.1	42.9	0.27
Nuts & seeds	47.2	41.7	11.1	54.3	35.2	10.5	0.57
Alcoholic drinks	80.6	15.7	3.7	66.6	28.6	4.8	0.07
Sugary beverages	89.8	9.3	0.9	89.5	7.6	2.9	0.54

* *p* of χ^2^ test.

**Table 3:** Before-after analysis by randomization group (213 women).

Variables	Intervention (110)			Control (103)		
	Baseline Mean ± SD	Six Months Mean ± SD	*p* *	Baseline Mean ± SD	Six Months Mean ± SD	*p* *
Weight (kg)	61.9 ± 10.9	60.0 ± 10.8	<0.001	64.1 ± 13.9	63.7 ± 13.7	0.19
BMI (kg/m^2^)	24.0 ± 4.8	23.3 ± 4.7	<0.001	24.2 ± 4.9	24.1 ± 4.7	0.15
Fat mass (%) Fat mass (kg) Lean mass (kg) Muscle mass (kg) Waist circ. (cm)	29.2 ± 7.9 18.5 ± 8.2 42.6 ± 3.4 40.3 ± 3.2 76.4 ± 9.8	27.5 ± 8.1 17.1 ± 8.0 42.2 ± 3.6 39.6 ± 4.5 74.3 ± 9.4	0.002 <0.001 0.17 0.11 <0.001	29.1 ± 9.1 19.5 ± 10.1 43.6 ± 4.6 41.4 ± 4.5 77.5 ± 12.2	29.6 ± 9.9 19.4 ± 10.2 43.3 ± 4.7 40.8 ± 5.7 76.3 ± 11.4	0.41 0.92 0.19 0.14 0.01
Hip circ. (cm)	100.2 ± 11.0	97.6 ± 8.9	<0.001	101.5 ± 9.3	100.7 ± 9.3	0.01
Systolic pressure (mmHg)	129.2 ± 18.7	125.1 ± 14.1	0.01	127.8 ± 14.0	124.1 ± 13.2	0.003
Diastolic pressure (mmHg)	82.2 ± 11.8	81.7 ± 9.2	0.64	81.9 ± 9.1	81.3 ± 8.1	0.51
Glycemia (mg/dL)	111.7 ± 21.4	102.2 ± 19.4	<0.001	109.0 ± 23.9	97.3 ± 17.9	<0.001
Total cholesterol (mg/dL)	203.4 ± 37.9	192.7 ± 34.2	<0.001	201.8 ± 39.0	197.3 ± 35.5	0.14
HDL cholesterol (mg/dL)	68.9 ± 16.5	68.6 ± 15.2	0.72	72.4 ± 19.0	73.1 ± 18.1	0.59
LDL cholesterol (mg/dL)	112.5 ± 32.8	103.9 ± 29.3	<0.001	111.0 ± 32.5	103.6 ± 28.7	0.01
Triglycerides (mg/dL)	110.8 ± 84.3	99.3 ± 48.6	0.08	93.6 ± 46.9	104.5 ± 56.5	0.004
IGF-I (ng/mL)	172.1 ± 72.1	157.1 ± 72.7	<0.001	171.1 ± 63.9	168.6 ± 60.5	0.57
Insulin (µIU/mL)	28.9 ± 19.8	18.4 ± 12.7	<0.001	24.7 ± 17.2	18.6 ± 12.3	0.001
IGFI-BP3 (µg/mL)	4.3 ± 1.1	4.4 ± 1.0	0.51	4.5 ± 1.0	4.3 ± 0.9	0.01

* *p* of *t*-test for difference between baseline and six months by group. BMI: Body Mass Index; HDL:High-Density Lipoprotein; LDL: Low Density Lipoprotein; IGF-I: Insulin-like Growth Factor I; IGFI-BP3: Insulin-like Growth Factor Binding Protein 3.

**Table 4:** Intention-to-treat analysis for ΔIGF-I, ΔIGFI-BP3, ΔIGF-I/IGFI-BP3 and Δinsulin.

Δ Variables	Intervention (110)	Control (103)	*p* *	*p* **
IGF-I (ng/mL)	−15.0	−2.5	0.02	0.02
IGFI-BP3 (µg/mL)	+0.06	−0.24	0.03	0.02
IGF-I/IGFI-BP3	−8.8	+0.75	0.04	0.04
Insulin (µIU/mL)	−10.5	−6.4	0.09	0.13

* *p* of ANOVA controlling for quintiles of age and BMI at baseline. ** *p* of ANOVA also controlling for quintiles of weight change.

**Table 5:** Proportion of women who improved, worse or did not change food consumptions between baseline and end of the six-months dietary intervention.

Food Frequency Consumption (%)	Intervention Group (110)			Control Group (103)			
	Worsening	No Change	Improvement	Worsening	No Change	Improvement	*p* *
Added sugars	%	%	%	%	%	%	
	11.1	57.4	31.5	16.2	58.1	25.7	0.44
Refined food	13.0	37.0	50.0	18.1	57.1	24.8	0.01
Whole-grain products	18.5	29.6	51.9	23.8	45.7	30.5	0.01
Whole-grain cereals	12.0	40.7	47.3	13.3	58.1	28.6	0.02
Legumes/Soy	23.2	50.9	25.9	20.0	64.8	15.2	0.08
Dairy products	2.7	51.9	45.4	15.3	49.5	35.2	0.01
Meat	25.0	50.0	25.0	19.1	51.4	29.5	0.52
Animal products	10.2	43.5	46.3	21.9	41.9	36.2	0.04
Vegetables	13.9	71.3	14.8	16.2	68.6	15.2	0.88
Fruits	28.7	47.2	24.1	24.8	47.6	27.6	0.75
Nuts & seeds	22.2	50.9	26.9	21.9	59.1	19	0.35
Alcholic drinks	8.3	81.5	10.2	14.3	68.6	17.1	0.09
Sugary beverages	0	90.7	9.3	3.8	89.5	6.7	0.10

* *p* of χ^2^ test to compare the frequency of women in the two randomized group according to their Δfood consumptions.
